# Mindfulness-Based Stress Reduction, Cognitive Behavioral Therapy, and Acupuncture in Chronic Low Back Pain: Protocol for Two Linked Randomized Controlled Trials

**DOI:** 10.2196/37823

**Published:** 2022-09-27

**Authors:** Sean Mackey, Gadi Gilam, Beth Darnall, Philippe Goldin, Jiang-Ti Kong, Christine Law, Marissa Heirich, Nicholas Karayannis, Ming-Chih Kao, Lu Tian, Rachel Manber, James Gross

**Affiliations:** 1 Division of Pain Medicine Department of Anesthesiology, Perioperative and Pain Medicine Stanford University Palo Alto, CA United States; 2 The Institute of Biomedical and Oral Research Faculty of Dental Medicine Hebrew University of Jerusalem Jerusalem Israel; 3 Betty Irene Moore School of Nursing University of California, Davis Sacramento, CA United States; 4 Department of Biomedical Data Science Stanford University Palo Alto, CA United States; 5 Department of Psychiatry and Behavioral Sciences Stanford University Palo Alto, CA United States; 6 Department of Psychology Stanford University Palo Alto, CA United States

**Keywords:** mind-body therapies, chronic low back pain, nonpharmacologic treatments, neuroimaging

## Abstract

**Background:**

Nonpharmacologic mind-body therapies have demonstrated efficacy in low back pain. However, the mechanisms underlying these therapies remain to be fully elucidated.

**Objective:**

In response to these knowledge gaps, the Stanford Center for Low Back Pain—a collaborative, National Institutes of Health P01–funded, multidisciplinary research center—was established to investigate the common and distinct biobehavioral mechanisms of three mind-body therapies for chronic low back pain: cognitive behavioral therapy (CBT) that is used to treat pain, mindfulness-based stress reduction (MBSR), and electroacupuncture. Here, we describe the design and implementation of the center structure and the associated randomized controlled trials for characterizing the mechanisms of chronic low back pain treatments.

**Methods:**

The multidisciplinary center is running two randomized controlled trials that share common resources for recruitment, enrollment, study execution, and data acquisition. We expect to recruit over 300 chronic low back pain participants across two projects and across different treatment arms within each project. The first project will examine pain-CBT compared with MBSR and a wait-list control group. The second project will examine real versus sham electroacupuncture. We will use behavioral, psychophysical, physical measure, and neuroimaging techniques to characterize the central pain modulatory and emotion regulatory systems in chronic low back pain at baseline and longitudinally. We will characterize how these interventions impact these systems, characterize the longitudinal treatment effects, and identify predictors of treatment efficacy.

**Results:**

Participant recruitment began on March 17, 2015, and will end in March 2023. Recruitment was halted in March 2020 due to COVID-19 and resumed in December 2021.

**Conclusions:**

This center uses a comprehensive approach to study chronic low back pain. Findings are expected to significantly advance our understanding in (1) the baseline and longitudinal mechanisms of chronic low back pain, (2) the common and distinctive mechanisms of three mind-body therapies, and (3) predictors of treatment response, thereby informing future delivery of nonpharmacologic chronic low back pain treatments.

**Trial Registration:**

ClinicalTrials.gov NCT02503475; https://clinicaltrials.gov/ct2/show/NCT02503475

**International Registered Report Identifier (IRRID):**

PRR1-10.2196/37823

## Introduction

An astounding 50 million to 100 million Americans live with ongoing pain, with approximately 20 million enduring high-impact chronic pain that includes substantially restricted work, social, and self-care activities [1-3]. Chronic low back pain is cited as the most common type of chronic pain condition [3].

Globally, chronic low back pain has an estimated prevalence of 10% to 20% in adults [4,5], is the leading cause of disability [6,7], and is one of the most clinically and economically burdensome medical conditions [3,8-10]. Despite the availability and increased use of traditional surgical, pharmacological, and physical treatments [11,12], the prevalence of chronic low back pain continues to increase at an alarming rate [6,13], and the health of individuals with chronic low back pain is deteriorating [3,14].

**Figure 1:**
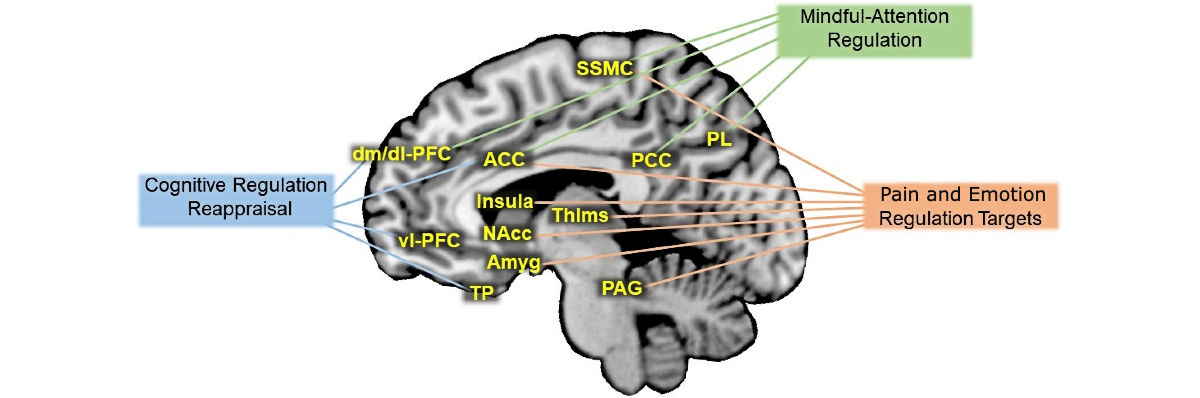
Schematic depiction of brain regions commonly involved in cognitive regulation reappraisal, mindful-attention regulation, and targets for pain and emotion regulation. ACC: anterior cingulate cortex; Amyg: amygdala; dm/dl-PFC: dorso medial and dorso lateral prefrontal cortex; NAcc: nucleus accumbens; PAG: periaqueductal gray; PCC: posterior cingulate cortex; PL: parietal lobe; SSMC: somatosensory motor cortex; Thlms: thalamus; TP: temporal pole; vl-PFC: ventro lateral prefrontal cortex.

Researchers have increasingly appreciated that chronic low back pain involves central nervous system abnormalities that cause, maintain, and amplify pain [15-20]. Acupuncture and other prominent “mind-body” treatments, such as cognitive behavioral therapy (CBT) and mindfulness-based stress reduction (MBSR), effectively engage participants in daily symptom self-management that impacts the central nervous system; these treatments offer low-risk, evidence-based, and economical therapies for this disabling condition [21-30]. However, there is a need to better characterize the underlying common and distinctive neurophysiological mechanisms of different mind-body treatments. Researchers can apply knowledge of these mechanisms to better optimize therapies targeted at an individual’s unique characteristics. Furthermore, this mechanistic information can serve as predictive biomarkers for whether a patient will be responsive to a particular mind-body therapy. A promising class of potential mechanisms relate to emotional reactivity and emotion regulation systems. Indeed, there is growing appreciation that these systems play a significant role in the perception, chronicity, and treatment efficacy of pain [17,31-35]. Research has established a clear link between emotion regulatory systems and pain perception in the brain (Figure 1) [36-46]. Importantly, CBT, MBSR, and acupuncture engage these same brain systems [28,29,47-52].

Pain-CBT is tailored to meet the specific needs of a person living with chronic pain. It includes pain education, relaxation training, cognitive restructuring, and behavioral interventions to target and modify maladaptive pain beliefs, reduce emotional reactivity, and increase adaptive emotion regulation strategies to enhance descending modulation of pain. Randomized controlled trials (RCTs) demonstrate the acceptability and short- and long-term efficacy of pain-CBT [30,53-61], and pain-CBT is a recommended first-line treatment for chronic low back pain [62].

MBSR targets early attentional processes to enhance an experiential approach toward the ongoing stream of thoughts, emotions, and sensations, through formal and informal meditation practices [63-65]. MBSR RCTs have demonstrated short- and long-term clinical benefits by improving physical function and health-related quality of life, and by reducing pain intensity, pain unpleasantness, and disability [30,53,54,66-72].

Based on traditional Chinese medicine, acupuncture consists of inserting fine metallic needles through the skin and into specific locations along pathways considered to be special conduits for electrical signals, to stimulate and restore the body’s “vital energy” [73-75]. A meta-analysis [76] and systematic reviews [30,77] concluded that acupuncture is effective for pain reduction. Electroacupuncture uses small electric currents, passed between pairs of needles, to stimulate acupuncture points in a standardized fashion. Preclinical and animal studies suggest that electroacupuncture might be more effective at relieving chronic pain than manual acupuncture [78].

Although research and RCTs indicate a positive effect of these mind-body treatments, their common and distinct mechanisms, their relative treatment efficacy, and the suitability of specific treatment components to specific subgroups remain unclear [79]. In response to these knowledge gaps, the National Center for Complementary and Integrative Health (NCCIH) funded the Stanford Center for Low Back Pain (grant P01AT006651), which was established as one of two Centers of Excellence for Research on Complementary and Alternative Medicine (CERCs). The second CERC was awarded to the Massachusetts General Hospital (grant P01AT006663) [80-83]. Here, we provide an overview of the design and implementation of the Stanford Center for Low Back Pain and the two RCTs aimed at characterizing the mechanisms of chronic low back pain treatments.

## Methods

### Scientific Focus

The Stanford Center for Low Back Pain will conduct two linked RCTs to elucidate the underlying mechanisms of pain-CBT, MBSR, and electroacupuncture for chronic low back pain, with a specific focus on the intersection of pain modulatory and emotion regulatory systems.

Researchers have identified that emotions are subject to diverse regulatory processes [84-86], such as cognitive regulation and attention regulation (Figure 1). Cognitive regulation uses language-based reasoning strategies to reconstrue the meaning of an emotion-eliciting situation to up- or down-modulate features of emotion instantiated in the ventral emotion system. Attention regulation modifies alerting, orienting, and executive attention [87]. Attention regulation involves training to focus on a selected object while inhibiting irrelevant distracter stimuli. Cognitive regulation is thought to be an active mechanism in pain-CBT, whereas attention regulation is thought to be an active mechanism in MBSR. These regulatory processes form the basis for investigating the effects of mind-body therapies in these linked RCTs.

The first RCT extends and expands on previous RCTs by replicating the efficacy of pain-CBT and MBSR for chronic low back pain treatment [53,54,88] and assessing the behavioral and neural mechanisms underlying their impact on pain. We will elucidate how these therapies differentially enhance the behavioral and neural indices of cognitive regulation and attention regulation during evoked pain, prospectively, and in comparison with wait-list (WL) participants with chronic low back pain that undergo no treatment.

The second RCT will evaluate the mechanisms underlying electroacupuncture treatment of chronic low back pain. We will compare how verum (ie, real) and sham electroacupuncture for chronic low back pain impact temporal summation and conditioned pain modulation, as measures of ascending pain facilitation and descending pain inhibition, respectively (see Kong et al [89] for specific trial protocol). As with the first RCT, we will characterize the impact of electroacupuncture on emotion regulatory processes using cognitive regulation and attention regulation during evoked pain. We expect the scientific knowledge gained by this CERC to translate to improved pain intervention for chronic low back pain.

### Stanford Center for Low Back Pain Organization

We organized the Stanford Center for Low Back Pain to promote cross-project collaborations, maintain recruitment consistency, enhance synergies across projects, provide common assessments and analyses, and optimize resource sharing. To facilitate administration, study execution, data collection, and analyses, the multidisciplinary projects will be supported by an Administrative Core, Clinical Research Core, Behavioral Core, and Neuroimaging and Psychophysics Core. The Administrative Core provides logistical and scientific coordination among the Scientific Cores and related individual projects. The Clinical Research Core supports scientific collaboration by focusing on regulatory submissions and oversight, participant recruitment and preliminary screening, participant safety, centralized data management and biostatistics, and education about human research. The Clinical Research Core will be the first contact for recruitment and preliminary screening of interested participants. This core will internally monitor recruitment progress, support data quality assurance, and perform between-project analyses to develop overall predictive models. The Behavioral Core will administer all behavioral measures (eg, validated questionnaires on pain, function, mood, expectancy, and quality of life) and oversee the pain-CBT and MBSR therapies. The Neuroimaging and Psychophysics Core will support all projects with a common battery to capture critical outcome measures administered by trained personnel using specialized psychophysics equipment. The battery consists of psychophysics (eg, evoked pain, temporal summation, and diffuse noxious inhibitory control) components. The neuroimaging aspect of the core will support acquisition, storage, and analysis of the structural and functional neuroimaging data.

### Study Design

#### Overview

The study includes two thematically related, prospective, single-center RCTs. Trial 1 compares pain-CBT versus MBSR versus no treatment; trial 2 compares verum (ie, real) versus sham electroacupuncture. While the RCTs will assess and compare treatment efficacies, the primary goals are to elucidate the underlying mechanisms of the therapies.

#### Specific Aims

##### Overview

The aims and hypotheses from the original proposal are described below. We anticipate that this mechanistic study will generate many additional aims and hypotheses using the comprehensive baseline and treatment data.

##### Aim 1: Assess Immediate and Longer-Term Impact of Pain-CBT Versus MBSR

We will compare pain-CBT–related and MBSR-related improvements in pain symptom severity and well-being in participants with chronic low back pain to each other and to WL-MBSR or WL-CBT participants (1) immediately posttreatment and to each other and (2) at 6 months posttreatment.

Hypothesis 1 is as follows: immediately posttreatment, we expect that both pain-CBT and MBSR will yield greater improvements in pain symptom severity and well-being in chronic low back pain participants compared to WL-MBSR and WL-CBT participants. We expect equivalent improvement for MBSR and pain-CBT immediately and at 6 months posttreatment.

##### Aim 2: Examine Pain-CBT Versus MBSR Treatment-Related Changes in Cognitive Regulation and Attentional Regulation

We will investigate whether pain-CBT and MBSR differentially enhance behavioral and neural indices of the ability to implement cognitive regulation and attention regulation during evoked low back pain in participants with chronic low back pain.

Hypothesis 2 is as follows: we expect treatment-specific improvements for pain-CBT and MBSR from pre- to posttreatment, as follows: (1) pain-CBT will improve cognitive regulation but not attention regulation and (2) MBSR will improve attention regulation but not cognitive regulation.

##### Aim 3: Examine Whether Changes in Cognitive Regulation and Attention Regulation Mediate Effects of Pain-CBT and MBSR

We will test whether cognitive regulation and attention regulation changes during treatment and posttreatment mediate pain symptoms and well-being at 6 months posttreatment.

Hypothesis 3 is as follows: we expect that improvement in cognitive regulation will mediate pain-CBT but not MBSR outcomes, and that improvement in attention regulation will mediate MBSR but not pain-CBT outcomes.

##### Aim 4: Characterize Primary Pain Regulation as a Mediator of Reduction in Back Pain Bothersomeness in Response to Treatment (Primary Clinical Outcome)

Hypothesis 4 is as follows: (1) real versus sham electroacupuncture will lead to greater reduction in temporal summation from baseline to the end of week 4 (ie, after 8 biweekly treatment sessions) and (2) change in temporal summation from baseline to week 4 will mediate reduction in back pain bothersomeness over the treatment course (ie, baseline to posttreatment, around week 10).

##### Aim 5: Assess Expectation of Benefits (Primary Psychological Measure) as a Moderator of Reduction in Back Pain Bothersomeness in Response to Treatment (Primary Clinical Outcome)

Hypothesis 5 is as follows: participants’ expectations of benefits will predict reduction in back pain bothersomeness scores during the treatment period.

Additionally, in the Discussion section, we present several secondary aims and deliverables resulting from this rich data set.

#### Study Sample and Setting

Across both studies, we aim to enroll more than 300 adults with chronic axial low back pain without radicular symptoms. This sample size accounts for an expected 30% attrition and provides sufficient statistical power for each project. An additional 30 healthy adults will be enrolled for a single neuroimaging visit as the healthy control group. Study screening, enrollment, pre- and posttreatment assessments and procedures, and the pain-CBT and MBSR treatment sessions will take place at the Stanford Systems Neuroscience and Pain Lab of the Stanford Division of Pain Medicine in Palo Alto, California. Verum or sham electroacupuncture sessions will take place in one of 10 acupuncture offices located across the San Francisco Bay Area, based on proximity to each participant. Magnetic resonance imaging (MRI) will occur at the Stanford Lucas Center for Imaging.

#### Inclusion and Exclusion Criteria

Recruitment will include 21- to 65-year-old men and women with chronic low back pain as determined by the National Institutes of Health (NIH) Task Force on Research Standards for chronic low back pain [90]. Tables 1 and 2 list inclusion and exclusion criteria, respectively, and describe how the criteria will be ascertained. A healthy control group will be recruited consisting of adults with no chronic pain and no medical or mental health condition that would interfere with study procedures. The control group will be age matched (ie, ±2 years) and gender matched to the chronic low back pain group at baseline.

**Table 1:** Inclusion criteria.

Inclusion criteria	Rationale	Sources
Axial low back pain as primary pain complaint without radicular symptoms	Study restricted to low back pain	A^a^, TS^b^, S^c^
Pain duration ≥3 months and pain experienced on at least half the days in the past 6 months	As per recent NIH^d^ Task Force on Research Standards for Chronic Low Back Pain [90]	A, TS, S
Average pain intensity ≥3/10 for the past 2 weeks after consent	No significant change in level of back pain	A, TS, S
Average pain intensity ≥4/10 for the past month at screening visit	Significant level of back pain to treat and to detect improvement	A, TS, S
English fluency	N/A^e^	A, TS, S
Males and females, 21 to 65 years of age	N/A^e^	A, TS, S

^a^A: automated data gathered from REDCap (Research Electronic Data Capture) surveys.

^b^TS: telephone screening.

^c^S: screening visit.

^d^NIH: National Institutes of Health.

^e^N/A: not applicable; this criterion was also applied to the healthy control group.

**Table 2:** Exclusion criteria.

Exclusion criteria	Rationale	Sources
Previous CBT^a^ or MBSR^b^ treatment or similar coursework in the last 2 years, or previous acupuncture treatment for any reason in the past 5 years, respectively, for the two projects^c^	Possible bias due to prior exposure to treatment	A^d^, TS^e^, S^f^
For the CBT and MBSR project, regular meditation practice (≥2 times/week, ≥15 minutes per meditation session, for ≥6 months) over the last 2 years^c^	Possible bias due to prior exposure to some essential aspects of MBSR	A, TS, S
Participating in any interventional research study or completed participation in the last 2 months; enrollment in an observational study is acceptable^c^	Treatment interference	A, TS, S
MRI^g^ contraindications (eg, metal implants and claustrophobia)^c^	MRI safety	A, TS, S
Neurologic disorder, history of seizures, stroke, or brain abnormalities, at the discretion of the study team^c^	Brain integrity interference	A, TS, S
Any radicular symptoms or other comorbid pain syndrome	Study restricted to low back pain	A, TS, S
Any medical condition (eg, active infection and heart disease) that would interfere with study procedures, at the discretion of the study team^c^	Medical conditions may confound mechanistic inferences	A, TS, S
Mental health conditions or treatment for mental health problems that would interfere with study procedures, at the discretion of the study team^c^	Mental health conditions may confound mechanistic inferences	A, TS, S
Medications: starting new medical treatment or medication for pain 2 months prior to initiation of study procedures; opioids ≥60 mg morphine equivalent units/day, anticonvulsants, benzodiazepines, beta-blockers, some antipsychotics, diabetic medications, or other medications that may interfere with study procedures at the discretion of the study team. TCAs^h^, gabapentinoids, SSRIs^i^, and SNRIs^j^ are *not* exclusionary if on a stable dose of at least 2 months^c^	Medications may confound mechanistic inferences	A, TS, S
Ongoing legal or disability claim or worker’s compensation (permanent and stationary disability not exclusionary)^c^	Ongoing legal or disability claims may confound mechanistic inferences	A, TS, S
Currently pregnant or planning to become pregnant^c^	Pregnancy may confound mechanistic inferences	A, TS, S
Disorders indicated by the MINI^k^ self-report questionnaire will be characterized and participants may be excluded at the discretion of the researcher	Condition that would make it difficult for a person to partake in treatments (eg, suicidality or psychotic disorders)	S

^a^CBT: cognitive behavioral therapy.

^b^MBSR: mindfulness-based stress reduction.

^c^This criterion was also applied to the healthy control group.

^d^A: automated data gathered from REDCap (Research Electronic Data Capture) surveys.

^e^TS: telephone screening.

^f^S: screening visit.

^g^MRI: magnetic resonance imaging.

^h^TCA: tricyclic antidepressant.

^i^SSRI: selective serotonin reuptake inhibitor.

^j^SNRI: serotonin and norepinephrine reuptake inhibitor.

^k^MINI: Mini–International Neuropsychiatric Interview.

#### Study Procedures

**Figure 2:**
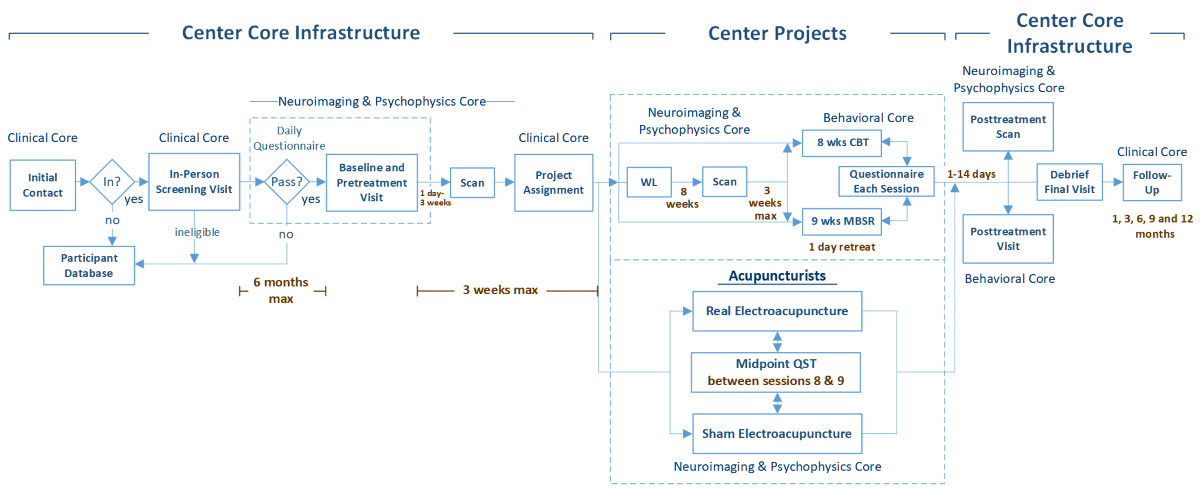
Flowchart overview of participant process in the Stanford Center for Low Back Pain project. btw: between; CBT: cognitive behavioral therapy; MBSR: mindfulness-based stress reduction; mo.: month; QST: quantitative sensory testing; wks: weeks; WL: wait-list.

Following initial contact, consent, the in-person screening visit, and eligibility assessment, participants enter one of the two projects (ie, pain-CBT vs MBSR or verum vs sham acupuncture) based on eligibility and preference. Subsequently, after baseline and pretreatment behavioral and neuroimaging assessment (see Figure 2 for participant process), participants will be randomized within each project to a treatment arm (see details below in Randomization section). Immediately posttreatment, participants in both projects will undergo additional behavioral and neuroimaging assessments. Participants will subsequently receive questionnaire assessments 1, 3, 6, 9, and 12 months posttreatment.

#### Ethics Approval

The study protocols were approved for human subject research by the Stanford Institutional Review Board (reference Nos. 22436 and 11689).

#### Recruitment and Screening

Participants will be recruited from social media marketing, the Stanford Systems Neuroscience and Pain Lab database, and local advertisements in clinics and in the community. All advertisements will direct interested individuals to complete a secure, online REDCap (Research Electronic Data Capture [91]) self-screening form for initial eligibility screening. Responding individuals will be contacted by phone for additional screening and, if potentially eligible, will be invited to an on-site consent and screening visit for further eligibility assessment. During this visit, the Mini–International Neuropsychiatric Interview (version 7.0) [92] will be administered, and additional study-specific criteria will be assessed (Tables 1 and 2). We will subsequently use experiential sampling methods, requiring each potential participant to provide daily feedback on pain symptoms, emotional functioning, and general health and well-being for a 3-week period after consent. Participants who do not adequately provide daily feedback will be excluded prior to randomization.

#### Randomization

Eligible participants will be assigned to a project based on current recruitment needs and randomized within each project separately. For example, to maximize participant retention in the pain-CBT and MBSR groups we prioritize recruitment for that cohort several weeks before the start of the treatment course. If a participant is unwilling to participate in a particular project (ie, pain-CBT or MBSR or electroacupuncture), they will be assigned to the other.

For the pain-CBT an MBSR project, participants will be randomized at the group level (ie, pain-CBT, MBSR, WL–pain-CBT, or WL-MBSR) and sequentially assigned to a cohort, based on when they complete their baseline assessment, without participants or assessors knowing the assigned arm. Given that our target treatment group size is 10 to 15 participants, we will allocate up to 18 participants per class to account for participants who fail to show up for treatment and overall attrition.

For the acupuncture project, participants will be randomized to the verum or sham arm using a biased coin algorithm [93], an adaptive randomization process assuring participant similarity between arms in preselected characteristics. For example, each participant will be classified as having less or more extreme baseline pain severity, using 7 out of 10 as a cutoff. For example, the randomization algorithm will automatically adjust the randomization ratio from 50:50 probability of assignment to 40:60, such that a participant with extreme pain will be less likely randomized into the arm that has more participants with extreme pain.

#### Blinding

Treatment providers will not be blind to treatment allocation and will not be involved in outcome assessment. Assessors will be blind to the treatment arm at pre- and posttreatment, including follow-up assessments. In the acupuncture project, participants will be blinded [89]. In the pain-CBT and MBSR project, participants cannot be blinded to treatment; however, allocation will occur only after baseline measures are completed.

#### Study Treatments

Pain-CBT will be delivered in group format (10-18 participants) across eight 2-hour weekly sessions by doctoral-level psychologists. Study psychologists will be trained on the pain-CBT study protocol and supervised by BD, a senior psychologist and research team member. The pain-CBT treatment will follow the manualized and validated protocol specifically tailored for chronic low back pain [54,94,95]. It consists of psychoeducation about pain, goal setting, progressive muscle relaxation, activity scheduling, cognitive restructuring of pain cognitions and fear avoidance beliefs, exposure to feared physical activities, relapse prevention, and stress-coping skills. The protocol also provides participants with the following: (1) a participant workbook with relevant worksheets for home and in-class use, (2) two CDs with eight guided relaxation and imagery exercises for home use, and (3) Turk and Winter’s book, The Pain Survival Guide [96], for optional reading (see Table 3 for in-class curriculum details).

MBSR will be delivered in a similar group format (10-18 participants) by a certified instructor with extensive experience delivering MBSR in clinical trials. MBSR will consist of 10 sessions, including an introductory session, eight 2.5-hour weekly sessions, and an optional 1-day meditation retreat. MBSR consists of informal and formal mindful meditation exercises practiced in class and at home. Each class consists of guided meditations, gentle movement exercises, lectures, and group discussions. The course includes a daylong retreat weekend session (Table 4) within the last 3 weeks. Between classes, students engage in home practice through meditation CDs, homework assignments, and readings from the course textbook, Full Catastrophe Living by Kabat-Zinn and Hanh [97]. All participants will be provided with two meditation CDs, Full Catastrophe Living, and handouts for homework assignments (see Table 3 for in-class curriculum details).

Participants in the WL arms will undergo no intervention for 8 weeks and then be assigned to pain-CBT or MBSR, according to availability. The initial 8 weeks of the WL condition will provide control for habituation to the assessments and for nonspecific factors, such as self-monitoring and contact with the research team.

**Table 3:** Pain-CBT^a^ and MBSR^b,c^ in-class curriculum.

Session No.	Pain-CBT curriculum	MBSR curriculum
1	Welcome and introductions, including group rules, logistics, etc; CBT rationale; pain physiotherapy; relaxation rationale; importance of home practice; and diaphragmatic breathing	Introduction to program, foundations of mindfulness, more right with you than wrong, and introduction to body scan meditation
2	Goal setting, activation, and pacing (SMART^d^, rest-activity cycle, etc); red flags; coping with flare-ups and creating a flare-up plan; and 7-muscle group PMR^e^	Patience, working with perceptions, the wandering mind, and the STOP^f^ exercise
3	Role of thoughts and feelings in pain, introduction to CBT and terms, introduction to 3-column thought record, and 4-muscle group PMR	Nonstriving, introduction to awareness of breathing meditation, mindful lying yoga, and attention vs disattention
4	Evaluating and generating alternate thoughts, introduction to evidence gathering, introduction to 4-column thought record, and 4-muscle group PMR, no tension	Nonjudging, responding vs reacting, seeing our patterns, sitting meditation, standing yoga, and research on stress and stress hardiness
5	More on evidence gathering and alternate thoughts (more detail), working with thoughts review, and body scan	Acknowledgment, group reflections on halfway point, small and large groups, sitting meditation, and Qi Gong
6	Thought records review and walking body scan	Letting it be, skillful communication, avoiding difficulty vs entering and blending, lovingkindness meditation, and walking meditation
7	Review of skills, troubleshooting regarding thought records, pain and mood, pain core beliefs, and sleep tips	Sitting meditation, mindful movement, trust and self-reliance, learning how to practice on one’s own, and mindfulness in everyday life
8	Review of skills, “signs” of not using skills: creating a plan for maintaining gains and dealing with setbacks, termination and wrap-up, and guided imagery	Sitting meditation, mindful movement, the class never ends: practice for the rest of your life, and course review and group reflection

^a^CBT: cognitive behavioral therapy.

^b^MSBR: mindfulness-based stress reduction.

^c^The MBSR curriculum also includes an orientation pre-MBSR session and the daylong retreat.

^d^SMART: Specific, Measurable, Achievable, Realistic, and Time-bound.

^e^PMR: progressive muscle relaxation.

^f^STOP: Stop, Take a breath, Observe, Proceed.

**Table 4:** Outline of mindfulness-based stress reduction group’s daylong retreat.

Time	Activity
9:30 AM	Introductions
10 AM	Awareness of breathing
10:15 AM	Lying yoga
10:30 AM	Body scan meditation
11:15 AM	Walking meditation
11:45 AM	Sitting meditation
12:15 PM	Lunch and rest
1:30 PM	Yoga
2 PM	Sitting meditation
2:30 PM	Walking meditation
2:45 PM	Sitting meditation
3:10 PM	Walking meditation
3:25 PM	Lovingkindness meditation
3:45 PM	Group discussion (check out)
4:10 PM	Farewell

A licensed acupuncturist will deliver both real and sham electroacupuncture over an 8-week period and 16 sessions. Treatment will consist of standardized electroacupuncture administration, both in terms of point selection and stimulation level. Each session will last approximately 45 minutes, except the first session, which will last 90 minutes and include an initial clinical assessment by the acupuncturist. The real electroacupuncture session will include the use of 20 needles per session. Flexibility in point selection is built into the standardized electroacupuncture protocol to allow for up to 10 more needles in cases where the patient reports hip or buttock pain or if the patient does not experience at least 30% pain reduction after the first four sessions. The sham electroacupuncture will include the use of nonpenetrating needles [98] at non–meridian points (ie, away from the center of the back) to minimize potential physiological effects, and since previous studies indicated potential pain relief attributed to nonpenetrating touch at the location of pain [99,100]. As previously implemented [101], sham electroacupuncture will be conducted by connecting broken wires to the electrical stimulators. The full protocol describing acupuncturist selection and training as well as treatment parameters, including point selection and location, has been published [89].

#### Treatment Fidelity

The manualized treatment protocols in pain-CBT and MBSR provide structured content for every treatment session, with little room for therapist drift. To further ensure treatment fidelity, a checklist was created for each session of both treatments. These fidelity checklists contain the essential components of the respective treatment session. Treatment fidelity ratings will be conducted in real time during each pain-CBT or MBSR session by a trained research specialist familiar with the treatment they are rating. Each treatment provider is given the criteria that will be used for treatment fidelity.

For acupuncture, treatment fidelity ratings will be based on reviews of structured case report forms completed by the acupuncturists after each session and audio session recordings. The review will include a random sample of 10% to 15% of sessions provided to participants seen by each acupuncturist. To minimize potential drift from treatment protocol and optimize fidelity, there will be quarterly meetings of the acupuncturists with JTK, who has extensive experience in delivering electroacupuncture for pain. During these meetings, corrective feedback of protocol deviations will be discussed, as needed.

#### Data Collection, Management, and Quality Control

Table 5 [92,102-127] summarizes the questionnaires, domains assessed, and timing of administration. All questionnaires will be completed via a Health Insurance Portability and Accountability Act–compliant REDCap platform. Participants will receive email reminders to complete their surveys via a secure link. Participants will also undergo physical assessment, quantitative sensory testing, and neuroimaging at pre- and posttreatment. The research team will assess data quality every 6 months by plotting data range and checking for missing values on REDCap, without unblinding the treatment assignment. Adherence and retention will be promoted by appointment reminders through email, text, and phone the day before scheduled assessments. In these reminders, we communicate the expectation and importance of attending all intervention and assessment visits. Participant compensation is contingent on the number of sessions they complete.

**Table 5:** Schedule of measures administration.

Measurement	Pretreatment		Treatment (8 weeks)	Posttreatment	Follow-up month						Longitudinal surveys^a^		
	Screening	Baseline			1	3	6	9	12				
Demographics	✓^b^												
Medical history	✓												
Mini–International Neuropsychiatric Interview [92]	✓												
Childhood Trauma Questionnaire [102]		✓											
Marlowe-Crowne Social Desirability Scale [103]		✓											
Stanford Expectations of Treatment Scale [104]			✓										
Response Style Questionnaire [105]		✓		✓									
Trait Meta Mood Scale and Toronto Alexithymia Scale hybrid [106]		✓		✓									
Credibility Expectancy Questionnaire [107]				✓									
Satisfaction with treatment^c^				✓									
Working Alliance Inventory [108]				✓									
Daily questionnaire^d^		✓	✓	✓									
Anxiety Sensitivity Index [109]		✓		✓			✓		✓				
Attentional Control Scale [110]		✓		✓			✓		✓				
Cognitive Distortions Questionnaire [111]		✓		✓			✓		✓				
Emotion Regulation Questionnaire [112]		✓		✓		✓	✓	✓	✓				
Five-Facet Mindfulness Questionnaire [113]		✓		✓		✓	✓	✓	✓				
Implicit Theories of Emotion Scale [114]		✓		✓		✓	✓	✓	✓				
Positive and Negative Affect Schedule [115]		✓		✓		✓	✓	✓	✓				
Short-form McGill Pain Questionnaire [116]		✓		✓		✓	✓	✓	✓				
Patient Global Impression of Change [117]				✓		✓	✓	✓	✓				
Weekly questionnaire^e^			✓		✓	✓	✓	✓	✓				
Sleep bruxism^c^		✓		✓		✓	✓	✓	✓				
Body pain map [118]		✓		✓		✓	✓	✓	✓	✓			
NIH^f^ PROMIS^g^ [119] (mobility, social isolation, and upper-extremity scales)				✓	✓	✓	✓	✓	✓	✓			
NIH PROMIS (anger, anxiety, depression, fatigue, pain behavior, pain intensity, pain interference, physical function, sleep disturbance, and sleep impairment scales)		✓		✓		✓	✓	✓	✓	✓			
Back pain bothersomeness^c^		✓		✓		✓	✓	✓	✓	✓			
Chronic Pain Acceptance Questionnaire [120]		✓		✓		✓	✓	✓	✓	✓			
Fear-Avoidance Belief Questionnaire [121]		✓		✓		✓	✓	✓	✓	✓			
Pain interference with sexual activities		✓		✓		✓	✓	✓	✓	✓			
Pain Catastrophizing Scale [122]		✓		✓		✓	✓	✓	✓	✓			
Pain Self-Efficacy Questionnaire [123]		✓		✓		✓	✓	✓	✓	✓			
Perceived Stress Scale [124]		✓		✓		✓	✓	✓	✓	✓			
Roland-Morris Disability Questionnaire [125]		✓		✓		✓	✓	✓	✓	✓			
Self-esteem^c^		✓		✓		✓	✓	✓	✓	✓			
Satisfaction with Life Scale [126]		✓		✓		✓	✓	✓	✓	✓			
NIH PROMIS (global health scale)		✓				✓				✓			

^a^Longitudinal surveys will be administered to participants who have been discontinued or withdrawn from the study. These questionnaires will be delivered electronically at 2 weeks and at 1, 2, 3, 6, and 12 months following the completion of their baseline behavioral appointment.

^b^A checkmark indicates that the measure was administered at the indicated time point.

^c^This is a single-item measure.

^d^The daily questionnaire consisted of several single items assessing pain severity, various physical health factors, and emotional coping. The questionnaire was administered in 2-week periods at baseline, at the beginning of weeks 1 and 3 of treatment, and posttreatment.

^e^The weekly questionnaire consisted of a combination of the following validated measures: Five-Facet Mindfulness Questionnaire; Pain Catastrophizing Scale; Working Alliance Inventory; Pain Self-Efficacy Questionnaire; Fear-Avoidance Belief Questionnaire; PROMIS pain intensity, fatigue, sleep disturbance, sleep interference, depression, anxiety, and anger scales; Behavioral Activation for Depression Scale (short form) [127]; Chronic Pain Acceptance Questionnaire; and Positive and Negative Affect Schedule. Measures for assessing frequency and capability of using cognitive and attention regulation to modulate back pain were included, in addition to single-item questions on pain intensity and relaxation.

^f^NIH: National Institutes of Health.

^g^PROMIS: Patient-Reported Outcomes Measurement Information System.

#### Physical Assessment

Various behavioral tasks will assess objective physical functioning [128,129]. The tasks include measures of forward-bending range of motion, sit-to-stand speed, single-leg balance, back muscle endurance, 10-meter walking speed, and 2-minute walking endurance. Trained staff administer the tests and document participant performance.

#### Quantitative Sensory Testing

A two-point discrimination task measures participant’s tactile acuity via an established protocol [130]. Using a dolorimeter (FDK-10; Wagner Instruments), pressure pain threshold and tolerance will be measured at the bilateral trapezius muscle bed, thumb nail beds, and lumbar regions (1-inch lateral to midline of L4-L5 interspinous space, because participants tend to be more sensitive in the low back). Pain threshold, tolerance, and curve response to thermal heat pain will be measured using a Medoc Pathway machine (Ramat Ishay). Testing tasks will proceed from the least to most stimulating. The test location is initially on the left hand over the thenar eminence and rotated to the opposite hand for each subsequent thermal testing. Although different in location, our methods for obtaining the blunt pressure and thermal sensitivity measures are similar to the German Research Network on Neuropathic Pain protocol [131]. Based on our lab’s published protocol [132], dynamic quantitative sensory testing, including thermal temporal summation and followed by conditioned pain modulation, will conclude the sensory testing module.

#### Neuroimaging

Most chronic low back pain participants will undergo two MRI scans of their brain: one pretreatment and one posttreatment. Participants allocated to the WL group will undergo three scans: before the waiting period, after the waiting period, and after the subsequent delivered treatment. The healthy control group will undergo one scan only as a baseline comparison to the pretreatment and pre-WL scans in the chronic low back pain group.

The brain scans will include structural and functional neuroimaging protocols. Structural scans include high-resolution gray and white matter scans. Functional scans will include a 10-minute resting-state scan and a heat-pain regulation task. This task is an experimental paradigm using heat as an evoked pain stimulus, applied to the low back. Prior to the scan, participants are trained in cognitive regulation and attention regulation, which will be implemented during evoked pain inside the scanner. While in the scanner, participants are presented with visual cues on a screen instructing them to respond to a 10-second heat stimulus in three different ways. The *respond* cue (ie, pain reaction) instructs participants to focus on the pain and allow their mind to react to the pain normally. The *reframe* cue (ie, cognitive regulation) instructs participants to reinterpret the way they think about the heat stimulus to reduce their negative reactivity to the pain. The *observe* cue (ie, attention regulation) instructs participants to observe and attend all aspects of their overall experience and to try not to focus on anything in particular. A fourth condition, *rest* (ie, no pain), serves as a baseline control and is not paired with a heat stimulus. After each heat stimulus, participants will rate their pain intensity and unpleasantness with a button box on a visual analogue scale ranging from 0 (“no pain”) to 10 (“worst imaginable pain”). The functional MRI heat-pain regulation task consists of a random sequence of the four conditions (10 trials per condition; no condition permitted to occur more than twice in a row) over 17.5 minutes. Though participants are instructed that each temperature may be the same or different than the one before, each heat stimulus is the same temperature: a pain intensity rating of approximately 6 out of 10 as determined by a fine thresholding procedure prior to scanning with each individual participant.

Standard analytic approaches will be used to preprocess and analyze the neuroimaging data, using common software packages, such as FSL, SPM, or both. The steps and algorithms of such software continue to improve, so the precise approach will be determined upon completion of data accrual. However, we anticipate that the general approaches will be similar to those of previously published methods [17,84,133-135]. For example, analysis of structural data may include probabilistic tissue segmentation, rigid co-registration for detecting within-individual differences, and high-dimensional normalization for establishing between-subject differences [134]. Preprocessing and analysis of functional data will most probably include head motion correction, spatial smoothing, high-pass temporal filtering, and usage of various general linear models to conduct various comparisons between conditions and groups [84].

#### Sample Size Determination

We determined sample sizes for both projects to ensure adequate power to detect significant mechanistic differences associated with the treatment comparisons.

##### Pain-CBT Versus MBSR

To estimate sample size, we conducted power analyses based on effect sizes derived from results from our pilot studies using G*Power (version 3; Heinrich-Heine-Universität Düsseldorf). Behavioral and functional MRI studies resulted in similar medium effect sizes. For Aim 1 (ie, pre- to posttreatment), testing a 2-way interaction in a 3-group (CBT, MBSR, and WL) × 3-time (pretreatment, posttreatment, and 6-month follow-up) repeated-measures analysis of variance (ANOVA) for clinical symptoms and well-being measures, a power of 0.80 can be obtained based on a medium effect (Cohen *d*=0.5), α=.05, and 32 participants per group. For Aim 2 (ie, treatment effect on cognitive regulation and attention regulation), testing a 2-way interaction in a 2-group (CBT and MBSR) × 2-time (pretreatment and posttreatment) repeated-measures ANOVA for negative emotion to evoked pain, a power of 0.80 can be obtained based on a medium effect (Cohen *d*=0.5), α=.05, and 32 participants per group. We anticipate 6 out of 40 (15%) dropped participants for each arm.

##### Acupuncture: Real Versus Sham

We computed sample size based on detecting differential changes in temporal summation, the primary mediator. Our pilot study suggested an average change of 10 to 25 points of the 100-point visual analogue scale with an SD of 12 following acupuncture. Prior studies suggest no treatment effect in controls receiving sham acupuncture [136]. However, given the imprecision of the pilot study’s estimates of effect sizes, we have powered the study to detect a medium effect (*d*=0.56) with 50 participants in each arm, using a 2-tailed test with α=.05 and β=.8. If the point estimates from the pilot prove accurate, this project will be very well powered (98% power) to detect large effects (*d*=0.833). To account for approximately 20% attrition, we will enroll around 120 study participants.

### Statistical Considerations

#### Overview

Below, we outline analyses to address the hypotheses outlined in the original proposal. Given the project’s primary focus on mechanisms rather than efficacy, we will perform the primary analyses using longitudinal treatment data with the per-protocol sample. This sample will consist of participants who completed all 3 assessment sessions (ie, pretreatment, posttreatment, and midpoint) and 5 or more out of 8 pain-CBT or MBSR treatments (or ≥13 out of 16 acupuncture visits). During the study and after completion, we will propose additional secondary aims and hypotheses using baseline and longitudinal data on chronic low back pain and before conduct of the analyses. Some of these secondary aims are presented in the Discussion section. Future hypotheses focusing on baseline data will use all available data.

#### Aims 1 to 3: Pain-CBT Versus MBSR

To address the efficacy aim (Aim 1), we will compute change scores from baseline to *immediately posttreatment* and *6 months posttreatment* for pain severity and well-being. We will conduct paired *t* tests to examine differential between-group changes immediately (CBT vs WL, MBSR vs WL, and CBT vs MBSR) and 6 months posttreatment (CBT vs MBSR). To test whether pain-CBT and MBSR differentially enhance behavioral and neural indices of the ability to implement cognitive regulation and attention regulation during evoked low back pain (Aim 2), we will conduct 2-treatment (CBT and MBSR) × 2-regulation (cognitive regulation and attention regulation) × 2-time (baseline and posttreatment) repeated-measures mixed-effects models and within and between-group *t* tests on (1) pain ratings and (2) neural responses in ventral emotion-generative and dorsal emotion-regulatory brain regions. To test whether changes in cognitive regulation mediate CBT but not MBSR outcomes, and whether changes in attention regulation mediate MBSR but not CBT outcomes (Aim 3), we will implement the MacArthur mediator model. Finally, we will test whether, immediately posttreatment, both CBT and MBSR yield greater improvements in pain symptom severity and well-being in chronic low back pain compared to WL participants. We expect equivalent improvement for MBSR and CBT immediately and 6 months posttreatment. Post-WL participants will enter CBT or MBSR treatment. If between-group *t* tests of immediate versus posttreatment WL-CBT and posttreatment WL-MBSR groups, separately, show no difference in pain symptoms and well-being, then we will use all participants treated with CBT or MBSR to conduct an exploratory moderator analysis to investigate whether any baseline demographic (eg, age and education), clinical (eg, comorbidity, prior treatment, age of onset, and symptom severity), or emotion (eg, depression, anxiety, and affect or trait emotion regulation) variables reliably identify who will benefit from CBT or MBSR.

#### Aims 4 to 5: Real Versus Sham Acupuncture

To test whether real electroacupuncture versus sham electroacupuncture leads to a greater reduction in temporal summation from baseline to the end of week 4 (Aim 4), we will conduct a 2-tailed, 2-sample *t* test. To test whether reduction in temporal summation from baseline to week 4 mediates reduction in weekly back pain bothersomeness scores (from baseline to posttreatment), we will use a McArthur mediation analysis. We will determine significant treatment effects on the clinical outcome using a mixed-effects model, with weekly back-pain bothersomeness scores as the dependent variable and treatment assignment as the independent variable. We will include a subject-specific intercept to account for within-patient correlations. We will test a second mixed-effects model, with longitudinally measured back-pain bothersomeness scores as the independent variable. Treatment arm, reduction in temporal summation from baseline to week 4, and their interaction will be independent variables. A significant main effect for change in temporal summation or interaction between change in temporal summation and treatment arm will be evidence of mediation. To test whether participant expectations of benefits predict reduction in back-pain bothersomeness scores (weeks 0-10; Aim 5), we will use a mixed-effects regression analysis with weekly measured back-pain bothersomeness scores as the dependent variable and expectation, treatment assignment, and their interaction as independent variables.

## Results

Participant recruitment began on March 17, 2015, and will end in March 2023. Recruitment was halted in March 2020 due to COVID-19 and resumed in December 2021. The trials were registered in ClinicalTrials.gov (NCT02503475) on July 21, 2015. The initial protocols and subsequent revisions follow NCCIH guidelines.

## Discussion

This NCCIH CERC P01–funded research strategy represents a novel direction for pain research for investigating common and distinct mechanisms of three mind-body therapies for chronic low back pain. The common and distinct mechanisms of these three mind-body therapies can be elucidated by comparing data across projects sharing phenotyping and data collection systems. While these therapies have been previously studied, the current effort extends them in several more ways. Specifically, we will be focusing on the role of different pain modulatory and emotion regulatory systems (ie, cognitive regulation and attention regulation) and their interactions. Moreover, we use neuroimaging to reveal underlying neural mechanisms that will support the development of biomarkers and neural targets for neuromodulatory techniques. These novel approaches will allow investigators to better delineate the biopsychosocial basis of chronic low back pain and support a personalized approach for treatment efficacy.

Furthermore, the comprehensive approach to systemically phenotyping people with chronic low back pain (expected N>300) will address a wealth of clinically important questions. Our comprehensive, longitudinal data set will represent one of the largest ever collected for chronic low back pain, with the exception of the anticipated deep phenotyping data of chronic low back pain by the NIH Back Pain Consortium several years out. The recruitment of healthy controls (ie, individuals without chronic pain, in general, and without chronic low back pain, specifically) allows investigators to elucidate novel mechanisms that may be unique to chronic low back pain, or that define clinically distinct chronic low back pain subtypes. Indeed, there is significant interest in defining subtypes of chronic pain, and its trajectories, that represent high-impact chronic pain. Additionally, the daily assessment data will provide valuable information on chronic low back pain symptom variability and flares.

Together, these extensive data collection efforts will help address the original overarching study hypotheses (see Methods section) and the following secondary clinically significant aims to advance clinical care for patients with chronic low back pain:

1. Identify biopsychosocial subsets of individuals with chronic low back pain with differing underlying pathogenesis defining their symptom severity, and define a diagnostic biomarker to classify chronic low back pain subtypes.
2. Identify predictive biomarkers using comprehensive baseline patterns of collected biopsychosocial data, combined with brief daily trajectories, to accurately predict treatment responsive to pain-CBT, MBSR, and real or sham acupuncture.
3. Identify differences in baseline and longitudinal emotion regulation characteristics that distinguish baseline characteristics of symptom severity in chronic low back pain and mediate treatment response to mind-body therapies.
4. Identify baseline pretreatment efficacy expectations associated with improved treatment outcomes and with distinctive neural imaging patterns.
5. Characterize symptom flares and mediating information on response to mind-body therapies through daily assessments of pain symptoms, emotional functioning, general health, and expectancy.
6. Determine whether individuals with longer chronic low back pain symptom duration have greater symptom severity, less psychosocial functioning, and different biological mechanisms than those with shorter symptom duration.
7. Determine whether individuals with localized chronic low back pain have different symptoms and underlying pathogenesis than those with chronic overlapping pain conditions and can be clustered based on baseline and longitudinal data.
8. Determine whether adverse childhood experiences are associated with greater symptom severity and decreased quality of life in individuals with chronic low back pain.
9. Determine whether various demographic factors, such as sex differences, ethnic background, and socioeconomic status, can distinguish baseline and longitudinal trajectories of symptom severity, neural mechanisms, and treatment responsiveness.

Results from these studies will advance our understanding of the pathophysiology and clinical characteristics of chronic low back pain, elucidate the mechanisms responsible for treatment response to mind-body therapies, and improve future clinical efforts for risk and treatment stratification to optimize care for individuals suffering from chronic low back pain.

## Abbreviations

ANOVA: analysis of variance
CBT: cognitive behavioral therapy
CERC: Center of Excellence for Research on Complementary and Alternative Medicine
MBSR: mindfulness-based stress reduction
MRI: magnetic resonance imaging
NCCIH: National Center for Complementary and Integrative Health
NIDA: National Institute on Drug Abuse
NIH: National Institutes of Health
RCT: randomized controlled trial
REDCap: Research Electronic Data Capture
WL: wait-list
